# Radiofrequency hyperthermia promotes the therapeutic effects on chemotherapeutic-resistant breast cancer when combined with heat shock protein promoter-controlled HSV-TK gene therapy: Toward imaging-guided interventional gene therapy

**DOI:** 10.18632/oncotarget.11346

**Published:** 2016-08-17

**Authors:** Jingfeng Luo, Xiaotian Wu, Fei Zhou, Yurong Zhou, Tongchun Huang, Fei Liu, Guocan Han, Luming Chen, Weixian Bai, Xia Wu, Jihong Sun, Xiaoming Yang

**Affiliations:** ^1^ Department of Radiology, Sir Run Run Shaw Hospital, Zhejiang University School of Medicine, Hangzhou, Zhejiang, China; ^2^ Image-Guided Bio-Molecular Intervention Research, Department of Radiology, University of Washington School of Medicine, Seattle, Washington, USA

**Keywords:** radiofrequency hyperthermia, heat shock protein promoter, HSV-TK, gene therapy, drug resistance

## Abstract

**Objective:**

Gene therapy is a frontier in modern medicine. In the present study, we explored a new technique for the effective treatment of multidrug-resistant (MDR) breast cancer by combining fully the advantages of multidisciplinary fields, including image-guided minimally invasive interventional oncology, radiofrequency technology, and direct intratumoral gene therapy.

**Results:**

Combination treatment with P_HSP_-TK plus RFH resulted in significantly higher TK gene transfection/expression, as well as a lower cell proliferation rate and a higher cell apoptosis index, than those of control groups. *In vivo* validation experiments with MRI confirmed that combination therapy resulted in a significant reduction of relative tumor volume compared with those of control animals, which was supported by the results of histologic and apoptosis analyses.

**Materials and methods:**

The heat shock protein promoter (P_HSP_) was used to precisely control the overexpression of thymidine kinase (TK) (P_HSP_-TK). Serial *in vitro* experiments were performed to confirm whether radiofrequency hyperthermia (RFH) could enhance P_HSP_-TK transfection and expression in a MDR breast cancer cell line (MCF7/Adr). Serial *in vivo* experiments were then carried out to validate the feasibility of the new technique, termed interventional RFH-enhanced direct intratumoral P_HSP_-TK gene therapy. The therapeutic effect of combination therapy was evaluated by MRI and confirmed by subsequent laboratory correlation.

**Conclusions:**

This study has established “proof-of-principle” of a new technique, interventional RFH-enhanced local gene therapy for MDR breast cancer, which may open new avenues for the effective management of MDR breast cancers via the simultaneous integration of interventional oncology, RF technology, and direct intratumoral gene therapy.

## INTRODUCTION

Breast cancer is the most common malignancy in women worldwide, accounting for 29% of new cancer cases among women in 2015, with an estimated annual mortality rate of 15% in the United States [1]. Adjuvant chemotherapy can reduce the risk of metastatic recurrence after surgical removal of breast cancer. However, currently-available chemo-drugs are effective only in certain subgroups of patients with breast cancer because they target specific proteins within tumors (e.g., trastuzumab targets breast cancers with HER2 expression). In addition, multidrug resistant (MDR) breast cancer leads to recurrence at distant metastatic sites in a proportion of breast cancer patients after adjuvant chemotherapeutic treatment. Drug resistance to chemotherapy can be divided into two types: intrinsic and acquired. Intrinsic resistance comprises cancers that possess chemodrug resistance related factors before the initiation of chemotherapy. Acquired resistance denotes cancers that acquire chemotherapy resistance during chemotherapy after being initially chemodrug sensitive, This may be caused by mutations or the activation of compensatory signaling pathways [2].

Cancer cells can adapt and develop one or more chemotherapeutic resistance pathways, which can result in treatment failure. In addition, it is known that multidrug resistance leads to failure of chemotherapy in over 90% of metastatic breast cancer patients [3]. Once tumor cells become resistant to a single class of anticancer agent, resistance to other unrelated drugs is more likely to occur. Despite advances in chemotherapy of breast cancer, resistance to chemotherapy remains a major obstacle to effective chemotherapy. Thus, it is necessary to explore alternative therapeutic approaches for the effective treatment of MDR breast cancer.

Gene therapy is a frontier in modern medicine. To date, more than 1000 gene therapy clinical trials and applications have been completed or are in progress worldwide. Most of these clinical trials have focused on oncology [4–6]. Among different gene therapies, the Herpes Simplex Virus Thymidine Kinase (HSV-TK) gene has been recognized as a promising tool for oncologic gene therapy. The HSV-TK gene is a suicide gene that converts non-toxic ganciclovir (GCV) monophosphate to toxic ganciclovir triphosphate, thereby killing cancer cells [7]. In addition, overexpression of TK genes can result in a bystander effect, via which phosphorylated GCV is transferred from TK-expressing cancer cells to neighboring non-gene transferred cancer cells, resulting in the death of the neighboring cancer cells [8]. The efficacy of gene therapy is primarily dependent on the amount and duration of gene expression. Therefore, regulating the expression of HSV-TK in the target organ is critical for the success of gene therapy.

Heat shock protein (HSP) is a highly conserved polypeptide that plays a role in the response to a variety of stresses, such as hyperthermia, aging, oxidative stress, and metabolic challenge. HSP-70 is a subgroup of the HSP family that is inducible and shows negligible basal expression in various types of cells [9]. The expression of HSPs increases sharply in the presence of heat shock [10], and the HSP-70 promoter is therefore commonly used to establish heat-controlled gene expression systems [11–13]. Previous studies from our group and others have shown that adjuvant hyperthermia at approximately 41°C to 45°C can enhance the effects of different therapeutics in various cancers [14–16]. However, hyperthermia-enhanced therapies are limited by various factors, including the availability of devices for local heat delivery to the target, unsatisfactory penetration of transporting heat to the target, thermal injury to healthy tissues adjacent to the targets, and the toxicity and mutagenic potential of heating vehicles. These weaknesses restrict the clinical application of hyperthermia-enhanced cancer therapies [17, 18].

Recent rapid advances in cancer therapy include image-guided, minimally invasive interventional oncology techniques [19, 20]. Previous studies from our group indicated that a combination treatment with local heat-controlled chemotherapy and interventional radiofrequency hyperthermia (RFH) enhances the therapy of various types of malignancies [21–23]. These results encouraged us to develop a new oncologic therapy technique, employing interventional mild RFH for enhanced heat-controlled suicide gene therapy, to solve the problem of multidrug resistance in breast cancer patients.

## RESULTS

### *In vitro* experiments

**Figure 1:**
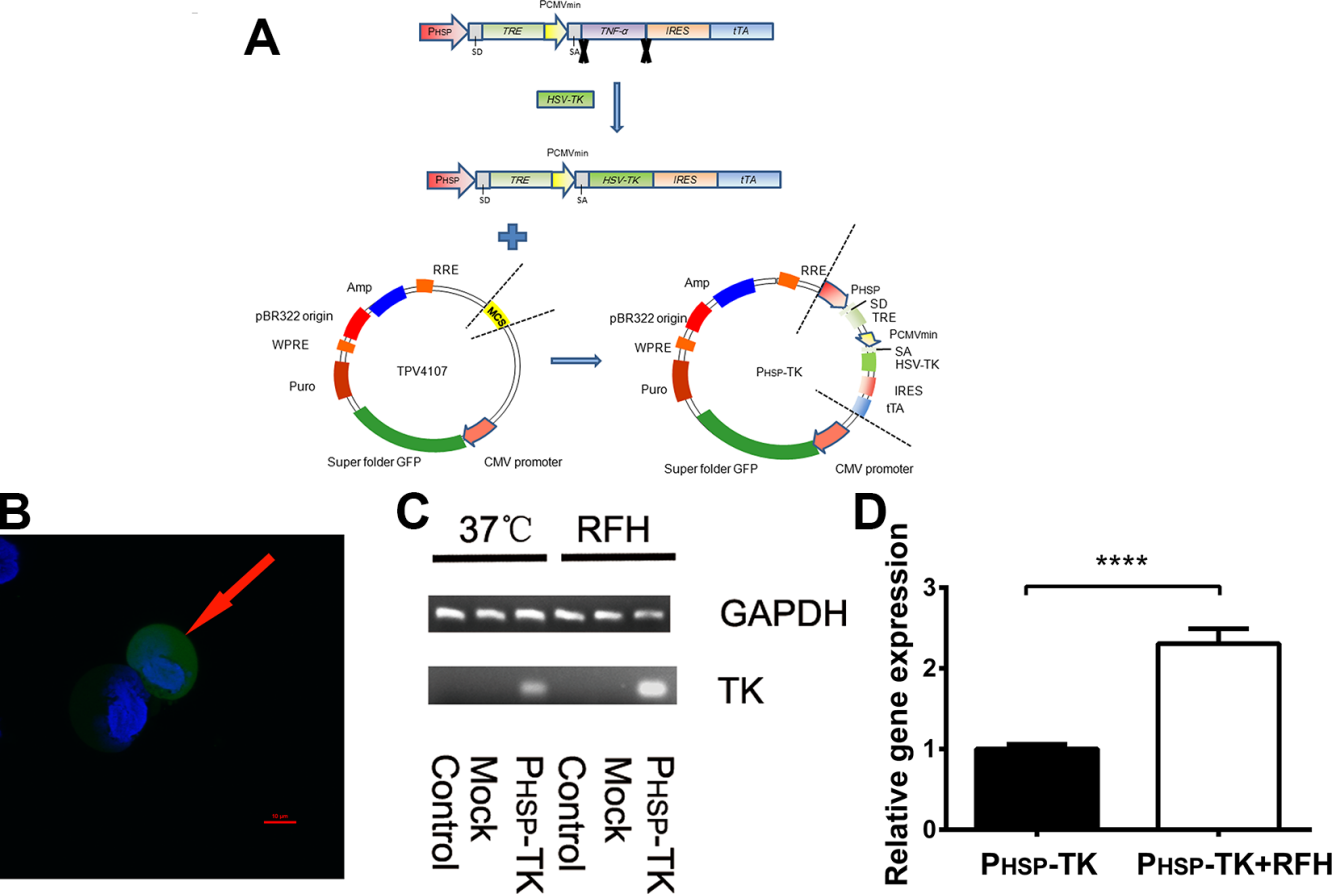
Construction of the PHSP-TK plasmid (**A**) The P_HSP_-TK plasmid was constructed and transfected into MCF7/ADR cells, which showed GFP florescence (arrow on **B**) (80× magnification). (**C**) RT-PCR further confirmed successful P_HSP_-TK gene expression at either 37°C or 45°C RFH. (**D**) The 45°C RFH condition significantly enhanced P_HSP_-TK gene expression compared with that in the P_HSP_-TK-only group, ****p < 0.0001.

The P_HSP_-TK plasmid was transfected into MCF7/Adr cells, and detection of GFP fluorescence indicated the successful expression of the P_HSP_-TK gene (Figure 1B). P_HSP_-TK gene expression was induced by RFH at 37°C and 45°C (Figure 1C). Real-time PCR demonstrated that RFH significantly enhanced P_HSP_-TK gene expression in MCF7/Adr cells (Figure 1D).

**Figure 2:**
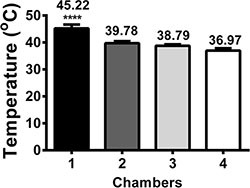
The formation of a stable temperature gradient from 37°C to 45°C The stable temperature gradient was recorded from 37°C to 45°C when chamber 1 was heated to 45°C.

When the RF generator was operated at 2–3 Watts through the MRIHG, the temperature in chamber 1 increased from 37°C to approximately 45°C, which generated a stable heat gradient along the four chambers (Figure 2).

**Figure 3:**
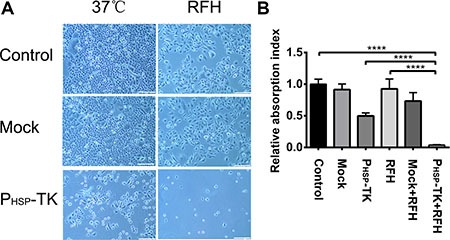
Results of *in vitro* experiments showing the cell phenotypes of MCF7/Adr after RFH-mediated gene therapy (**A**) The cell killing effect of combination treatment with P_HSP_-TK + RFH was greater than those of other treatments. (10× magnification). (**B**) Results of the CCK8 cell proliferation assay, showing a significantly lower cell survival in the combination treatment group with P_HSP_-TK + RFH than in the other cell groups (****p < 0.0001). Scale bars = 200 um.

**Figure 4:**
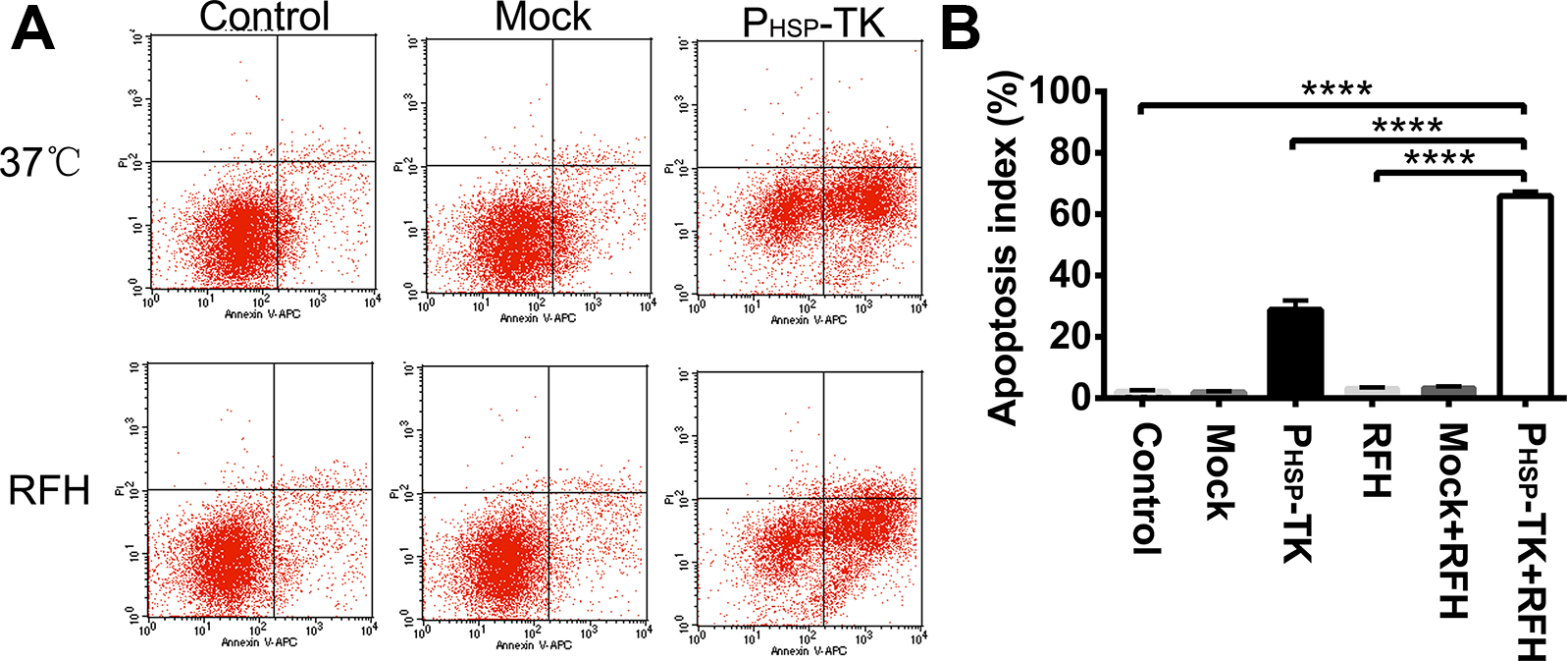
Representative results of the cell apoptosis assay with Annexin V-APC and PI double staining (**A** and **B**) Flow cytometric profiles and quantification, showing a higher percentage of apoptotic cells in the combination treatment with P_HSP_-TK + RFH group than in the other groups (****p < 0.0001).

RFH enhanced the cell killing efficacy of the P_HSP_-TK gene, resulting in a decrease in cell survival compared with other cell groups (Figure 3A). This was confirmed by performing cell proliferation assays, the results of which showed that combination treatment with P_HSP_-TK plus RFH significantly inhibited tumor cell proliferation, resulting in a lower cell viability rate (3.8% ± 0.2%) than that of the other treatment groups (100% ± 3.22% vs. 91.4% ± 3.7% vs. 49.8% ± 2.0% vs. 92.6% ± 6.4% vs. 73.3% ± 5.5%, Control, Mock, P_HSP_-TK, RFH, and RFH + Mock groups, *p* < 0.0001) (Figure 3B). Cell apoptosis assays showed that the combination treatment with P_HSP_-TK + RFH resulted in a higher rate of apoptosis (65.99% ± 0.78%) than other treatment groups (2.13% ± 0.27% vs. 1.99% ± 0.19% vs. 28.89% ± 1.72% vs. 3.25% ± 0.18% vs. 3.49% ± 0.21%, Control, Mock, P_HSP_-TK, RFH, and RFH + Mock groups, *p* < 0.0001) (Figure 4A and 4B).

### *In vivo* experiments

**Figure 5:**
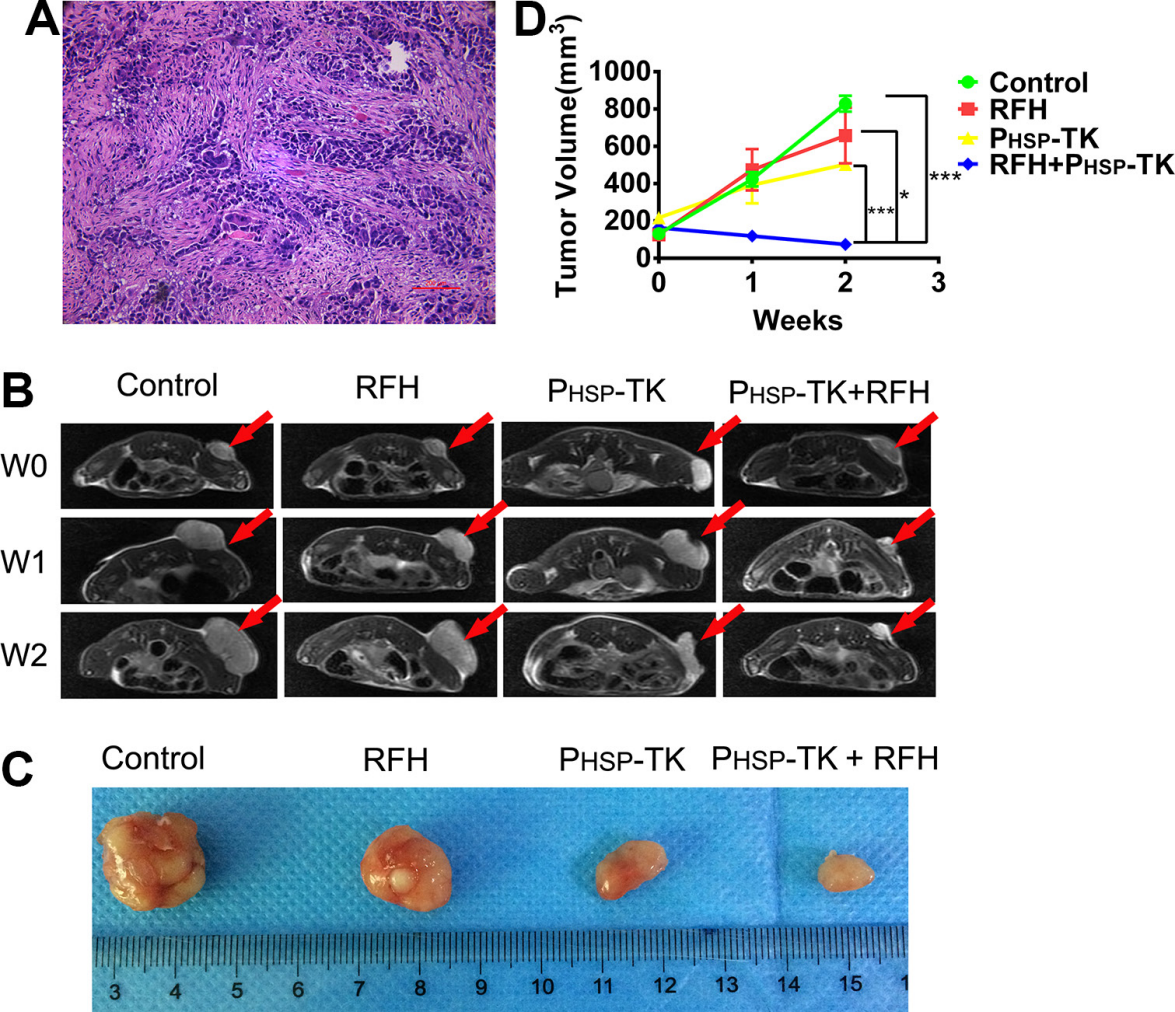
Results of *in vivo* experiments demonstrating tumor volume changes among the four animal study groups with different treatments (**A**) The successful creation of breast xenograft tumors was confirmed by pathological examination (20× magnification). (**B**) Representative MRI of four mouse models with different treatments on weeks 0, 1, and 2, showing a reduction of the tumor mass (arrow) in mice treated with P_HSP_-TK + RFH, compared with the control, RFH, and P_HSP_-TK groups. (**C**) Representative pathology results of tumor masses from different groups, demonstrating that the smallest tumor was obtained after combination treatment with P_HSP_-TK + RFH. (**D**) Quantitative analysis of relative tumor volumes among different animal groups with various treatments, showing that combination treatment with P_HSP_-TK plus RFH significantly inhibited tumor growth at week 2, in comparison with the other three animal groups (****p < 0.0001, *p < 0. 05).

Successful establishment of animal models bearing breast tumors was confirmed by histopathological examination (Figure 5A). MRI showed that tumors were smaller in mice treated with combination therapy (P_HSP_-TK + RFH) than in the control, RFH-only, or P_HSP_-TK-only treatment groups (Figure 5B). Representative images of excised tumors are shown in Figure 5C. The average RTV was significantly smaller in the P_HSP_-TK + RFH group (1.10 ± 0.29) than in the control, RFH, or P_HSP_-TK groups on day 14 after gene therapy (3.72 ± 0.72, 6.04 ± 1.52, and 7.27 ± 1.71, respectively; *p* < 0.05, *p* < 0.0001, and *p* < 0.0001; Figure 5D).

**Figure 6:**
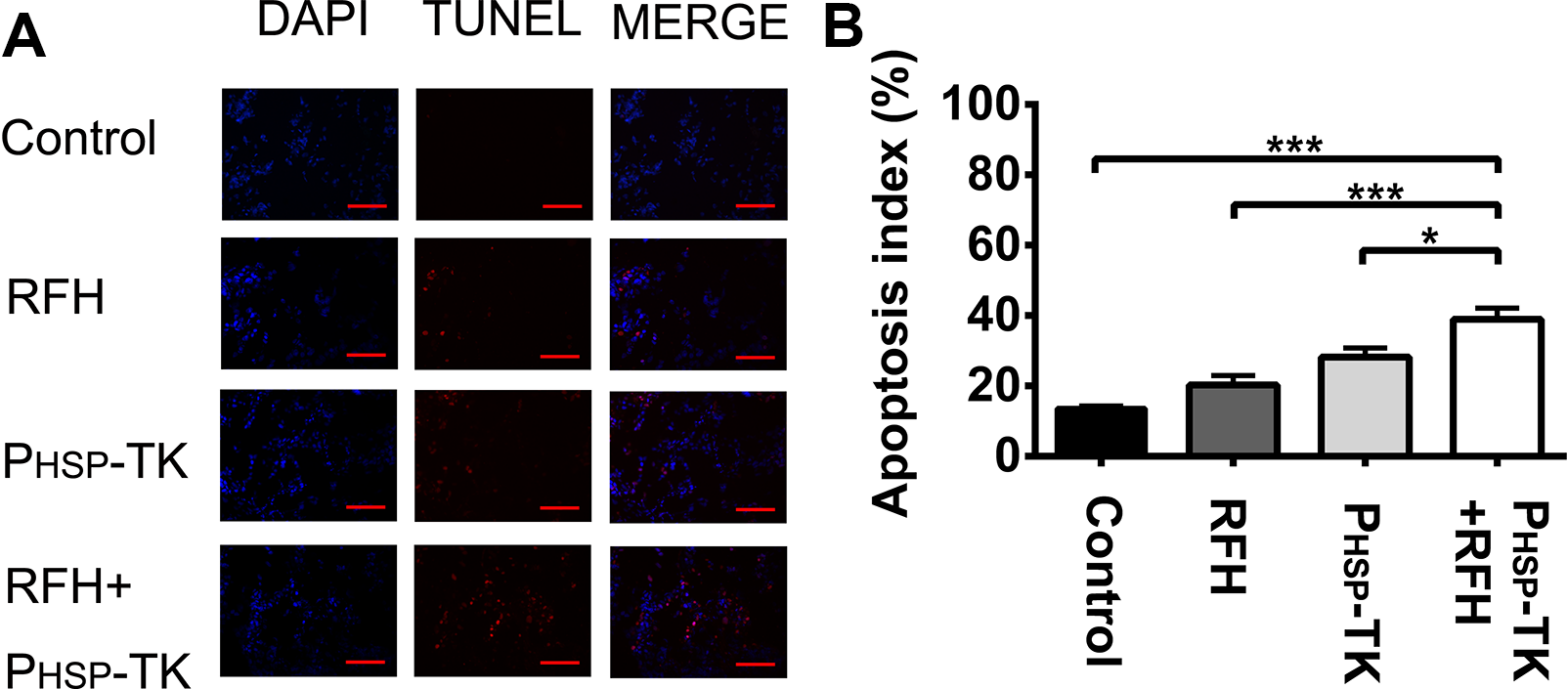
Apoptosis assay of tumor tissues (**A** and **B**) TUNEL staining and quantification of apoptosis index showing a greater number of apoptotic cells in the P_HSP_-TK + RFH treatment group than in other three animal groups (***p < 0.001, *p < 0.05). Scale bars = 100 um.

Subsequent laboratory examinations further confirmed that the number of apoptotic cells and average apoptosis index of the P_HSP_-TK + RFH group (38.94% ± 3.21%) were significantly higher than those of the control, RFH, or P_HSP_-TK groups (13.66% ± 0.64%, 20.30% ± 2.68%, and 28.17% ± 2.64%, respectively; *p* < 0.001, *p* < 0.001, and *p* < 0.05; Figure 6).

## DISCUSSION

Chemotherapy is one of the three principal treatments for breast cancer, namely surgery, chemotherapy, and radiotherapy. The resistance of tumor cells to anticancer agents results in treatment failure in breast cancer. Overcoming this obstacle has become an urgent issue for the effective management of patients with breast cancer. Gene therapy is becoming a promising option to the treatment of MDR breast cancer. Suicide gene therapy has been applied to different cancers, with some effectiveness [24–26]. However, some unresolved issues, such as the limited dose of systemically delivered genes and low level of gene expression at the targets *in vivo*, have limited the clinical application of cancer gene therapy. In this work, we attempted to solve these problems. We first constructed the heat shock protein promoter-mediated HSV-TK gene-carrying plasmid and transfected it into breast cancer cells. After RFH, HSV-TK gene expression was significantly increased (Figure 1C and 1D). Our study have confirmed that the proliferation of tumor cells was inhibited *in vitro*, and tumor volume was reduced *in vivo*, compared with control, RFH-only, or PHSP-TK-only treatment groups (Figure 5D). These results indicate that RFH can increase HSV-TK gene expression and thereby enhance the killing effects of suicide gene therapy on chemodrug resistant breast cancers. The potential mechanisms of RFH-enhanced gene expression include tissue fracture via heating, higher permeability of the cytoplasmic membrane, higher cellular metabolism, and activation of the HSP pathway [27–29]. These mechanisms facilitate the entrance of therapeutics into target tumor cells, thereby promoting the effective destruction of tumor tissue.

Temperature fluctuation can influence the efficiency of gene expression, so the heating temperature must be precisely controlled. Some groups have employed placing mice bearing tumor xenografts in preheated air [30] or a water bath [31] to promote the therapeutic effects. These conventional hyperthermia methods can result in non-uniform heating and irregular changes in temperature. To overcome this barrier, we placed a sensitive fiber optic temperature sensor into the tumor mass near the RFH antenna, which ensured that RFH at the target site could be precisely controlled.

Current technologies allow the delivery of therapeutic genes using a systemic approach, which often results in limited doses of therapeutic genes at the target. In addition, the systemic delivery of genes creates a risk of toxicity to healthy and vital organs, particularly when the amount of intravenously-administered therapeutic gene dose is increased to reach the target [32]. Image-guided minimally invasive interventional techniques provide the advantage of delivering highly concentrated genes locally and directly to targets without causing damage to healthy organs [33, 34]. Recently, some studies have confirmed that local hyperthermia at 40–45°C via an interventional approach can significantly enhance the effectiveness of different therapeutics, including genes, in various cancers [21, 35].

In the present study, we attempted to overcome the disadvantages of current gene therapy techniques by combining the advantages of (i) interventional techniques for direct intratumoral delivery of highly concentrated therapeutic genes to targets; and (ii) interventional RF-mediated local hyperthermia to enhance gene expression at the target sites. This combination treatment smoothed the way for the treatment of chemodrug-resistant breast cancers.

Although our results are encouraging, further studies to optimize the therapeutic protocol and long-term follow-up to evaluate the therapeutic effects of this novel combination are warranted.

## CONCLUSIONS

The present study confirmed that interventional RFH can promote HSP promoter-mediated TK suicide gene transfection and expression in chemotherapeutic-resistant breast cancers, thereby enhancing the efficacy of TK/GCV gene therapy. This technical development may open new avenues for the effective management of chemotherapeutic-resistant breast malignancies via the simultaneous integration of interventional oncology, RF technology, and direct intratumoral gene therapy, instead of systemic gene therapy.

## MATERIALS AND METHODS

The present study included two primary components: *in vitro* experiments were performed in MCF7/Adr to establish “proof-of-principle” for the RFH-enhanced killing effect of genes on multidrug-resistant human breast cancer cells; and *in vivo* experiments on living animals were used to validate the feasibility of the new technique, interventional RFH-enhanced direct intratumoral gene therapy for chemotherapeutic-resistant breast cancer.

### *In vitro* experiments

#### Construction of the P_HSP_-TK plasmid

The gene expression plasmid (PHSP-TRE/TNF-alpha/IRES/tTA) was kindly provided by Prof. Masamichi Kamihira (Kyushu University, Fukuoka, Japan). The promoter sequence of HSP and the TRE and IRES-tTA elements were from the plasmid PHSP-TRE_TNF-alpha_IRES_tTA, whereas the HSV-TK gene sequence was from the plasmid pQMSCV/PTNIG. The DNA fragments encoding the HSV-TK gene were inserted into the PHSP-TRE_TNF-α_IRES_tTA plasmid, replacing the TNF-α sequence. Then, the fragments were ligated into the lentivirus vector TPV4107, which included a green fluorescence protein (GFP) gene (Shengbo Company, Shanghai, China). The overall cloning process of the final therapeutic gene product, P_HSP_-TK, is shown in Figure 1A, and the DNA sequences were subsequently confirmed using a DNA sequencer. The lentiviral vector, TPV4107, was used as the mock vector, which did not include the P_HSP_-TK gene.

#### Preparation of lentivirus

Lentiviruses were produced and purified as previously described [36]. Lentiviral vectors were co-transfected into 293T cells to produce lentiviral particles with Lipofectamine 2000 reagents (Thermo Fisher, Waltham, MA, USA). After 16 h, the 293T cells were cultivated with fresh medium at 37°C in 5% CO_2_ incubator for 24 h. Collected and pooled supernatants were then centrifuged at 70,000 g for 2 h at 20°C. After discarding the supernatants, the viral pellets were resuspended in 100 ul of 1× HBSS and stored at −80°C for future use.

#### Cell culture

The doxorubicin-resistant human breast cancer line MCF7/ADR was kindly provided by Professor Jianqing Gao (College of Pharmacy, Zhejiang University). This human breast cancer cell line was used because the MCF7/Adr cell line is resistant to multi-chemodrugs (such as Doxorubicin, the first-line chemodrug for systemic treatment of breast cancer). The cells were cultured in Dulbecco's Modified Eagle's Medium (DMEM) supplemented with 10% FBS, 100 U/ml penicillin, and 100 mg/ml streptomycin (Thermo Fisher, Waltham, MA, USA). Cultured cells were maintained at 37°C in a humidified atmosphere containing 5% CO_2_.

#### Detection of P_HSP_-TK gene transfection and expression

MCF7/Adr cells were split into four-chamber plates (4 × 10^4^/chamber). After 24 h, the cells were incubated with P_HSP_-TK/lentivirus [multiplicity of infection (MOI) = 20] for 12 h, followed by the addition of fresh culture medium. Subsequently, qPCR was performed to determine HSV-TK gene expression at 72 h post-transfection. qPCR was performed using an ABI 7700 sequence detection system (Applied Biosystems, Foster City, CA, USA), and the amplifications were performed using SYBR Green PCR Master Mix (Toyobo, Osaka, Japan).

#### Real-time quantitative PCR

Total RNA was extracted with the Trizol reagent (Invitrogen, Carlsbad, CA, USA) following the manufacturer's instructions. cDNA synthesis was performed using 1 μg of total RNA. The thermal cycling conditions were as follows: 95°C for 5 min, 35 cycles at 95°C for 30 sec, 59°C for 30 sec, and 72°C for 45 sec. Glyceraldehyde 3-phosphate dehydrogenase (GAPDH) was used as an internal control to normalize the gene expression level. The relative quantification of gene expression was detected using the 2^-ΔΔCt^ method [37]. The primer sequences are shown in Table 1.

**Table 1:** Primer sequences used for mRNA quantification by qPCR

Gene	Forward primer sequence	Reverse Primer sequence
HSV1-TK	5′-AGAAAATGCCCACGCTACTG-3′	5′-GTAAGTCATCGGCTCGGGTA-3′
GAPDH	5′-GGAGCGAGATCCCTCCAAAAT-3′	5′-GGCTGTTGTCATACTTCTCATGG-3′

#### Radiofrequency hyperthermia

Equal numbers of breast cancer cells were seeded in each chamber of a four-chamber cell culture slide (Nalge Nunc International, Rochester, NY, USA) at a density of 5 × 10^4^ cells per chamber and incubated in a 37°C water bath (Supplementary Figure S1). The cells were divided into different groups and treated as follows: (i) gene transfection of P_HSP_-TK plus RFH; (ii) P_HSP_-TK gene transfection-only; (iii) RFH-only; (iv) gene transfection of Mock plus RFH; (v) Mock transfection-only; and (vi) no treatment as a control. For statistical analysis, each group included six chambers. For gene transfection, P_HSP_-TK/lentivirus (MOI = 20/chamber) was added to the cell-containing chamber for 48 h, followed by heating at 45°C for 20 min using an RF system. For the cell groups with RF heating, a 0.032 inch magnetic resonance imaging-heating-guidewire (MRIHG) was placed under the bottom of chamber, and then connected to a 2450 MHz RF generator (GMP150, OPTHOS, Rockville, MD, USA) to heat the slides at 45°C for 20 min. The temperature of the chambers was constantly recorded by a thermometer (Photon Control, Burnaby, British Colombia, Canada) (Supplementary Figure S1).

After the treated cells were incubated for 48 h, the culture medium of these cells was replaced by fresh culture medium containing GCV (10 μg/ml) for another 48 h.

#### Cytotoxic and cell apoptosis assays

Cytotoxic and cell apoptosis assays were performed to evaluate the cell killing effects of P_HSP_-TK/GCV on breast cancer cells. Cell proliferation was assessed using the Cell Counting Kit-8 (CCK-8, Dojindo, Japan) according to the manufacturer's protocol. Briefly, CCK-8 solution (100 μl) was added into the cell chamber and incubated for 2 h, and the absorbance of each chamber was measured at 450 nm using a Universal Microplate Reader (BIO-TEK Instruments, Minneapolis, MN, USA). Then, cell apoptosis assay was performed as described in the Annexin V-APC apoptosis detection kit using flow cytometry analysis (Becton Dickinson FACScan, Mount View, VA, USA). Then, the apoptosis index was analyzed by counting the number of Annexin V positive cells (red staining) per HPF (high-power field, 40×) in 10 slides for each group.

### *In vivo* experiments

#### RFH-mediated gene therapy

Animal experiments were approved by our Institutional Animal Care and Use Committee. Twenty-four 6-week-old BALB/c nude female mice (Slac Laboratory Animal Center, Shanghai, China) were divided into four subject groups (six mice per group), and treated as follows: (i) RFH + P_HSP_-TK; (ii) P_HSP_-TK-only; (iii) RFH-only; and (iv) phosphate-buffered saline (PBS) as a control. Human chemotherapeutic-resistant breast cancer cells (5 × 10^6^) were subcutaneously and unilaterally implanted into the back of mice to create animal models with breast cancers. Once the tumor grew to approximately 5 mm in diameter, 10–15 ul concentrated viruses were injected into tumors each time with a total of 6–10 injections per tumor. Thus, 1 × 10^8^ TU/100 μl P_HSP_-TK/lentivirus were directly injected into the tumor mass. Two days after the gene therapy, the 0.032 inch MRIHG was inserted into the gene-targeted tumor for local heating at 45°C for 20 min. A 2.7 mm micro-thermometry fiber was placed in parallel to the MRIHG, to instantly measure the temperature changes caused by RFH at the target tumor (Supplementary Figure S2). Subsequently, intraperitoneal GCV (25 mg/kg) was administered every 2 days for 14 days.

#### MRI

Mice were mechanically ventilated with 1–3% isoflurane mixed with 0.5 l/min oxygen for MRI follow-up. MRI was performed with a 3.0-Tesla MR scanner (GE Healthcare Corporation, Chicago, USA) by placing the mouse into a 100 mm-diameter micro-imaging coil. T2-weighted images (T2WI) were acquired by rapid acquisition with the following OAx T2 FSE spin echo sequence: TR/TE = 2660/80 ms, field of view = 8 cm, matrix = 256 × 256, section thickness = 1.5 mm, intersection gap = 0.5 mm, NEX = 2, and total scan time = 1 min and 51 sec. MRI was performed on days 0, 7, and 14 after the gene therapy. In this study, tumor growth was followed up for up to two weeks and combination therapy did not prolong overall survival after the treatments. This is because the human breast cancer xenografts developed in this study grew fast, posing the risk that those in the control group could reach a large size (over 10% body weight), which is not permitted by the IACUC in this study.

#### Histology confirmation

After achieving satisfactory MRI, the animals were euthanized and tumors were harvested. The volume (V) of each tumor mass was calculated with the following formula: V = A*B^2^/2 (where A is the longer diameter and B is the shorter one) [4]. Because of variation in tumor size, the relative tumor volume (RTV) was calculated with the following formula: RTV = V_n_/V_0_ (where V_n_ = tumor volume on day 7 or 14 post-treatment and V_0_ = tumor volume pre-treatment). Then, the tumor tissues were examined using different laboratory methods, including (i) hematoxylin and eosin (H&E) staining to confirm the formation of breast tumors; and (ii) terminal dUTP nick end labeling (TUNEL) to examine cell apoptosis. The number of apoptotic cells was counted by Image-Pro Plus 6 software (Media Cybernetics, Rockville, MD, USA).

#### Statistical analysis

Statistical analyses were performed by one-way ANOVA to compare *in vitro* gene expression rates, cell killing efficacies, and apoptosis indices. Two-way ANOVA was performed to compare *in vivo* tumor sizes among different subject groups at three time points. A *p*-value < 0.05 was considered statistically significant.

## SUPPLEMENTARY MATERIALS FIGURES


