# Supplementary material for: Radiofrequency hyperthermia promotes the therapeutic effects on chemotherapeutic-resistant breast cancer when combined with heat shock protein promoter-controlled HSV-TK gene therapy: Toward imaging-guided interventional gene therapy
Source: Oncotarget. 2016 Aug 17;7(40):65042–51. doi: 10.18632/oncotarget.11346 (PMC5323137; doi:10.18632/oncotarget.11346)
Supplement: Supplementary file 1 [file oncotarget-07-65042-s001.pdf]

Radiofrequency hyperthermia promotes the therapeutic effects on chemotherapeutic-resistant breast cancer when combined with heat shock protein promoter-controlled HSV-TK gene therapy: Toward imaging-guided interventional gene therapy

Supplementary Materials

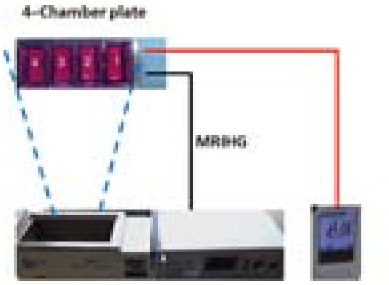

Supplementary Figure S1: *In vitro* experiment set-up for RFH-mediated gene therapy of human breast cancer cells. The four-chamber cell culture slide was positioned in the 37°C water bath. A 0.032 inch magnetic resonance imaging-heating-guidewire (MRIHG) was placed under the bottom of chamber 1, which resulted in a stable temperature gradient along the four-chamber slide when operating the RF generator. The RFH temperature of each chamber was precisely measured and controlled by the thermometer.

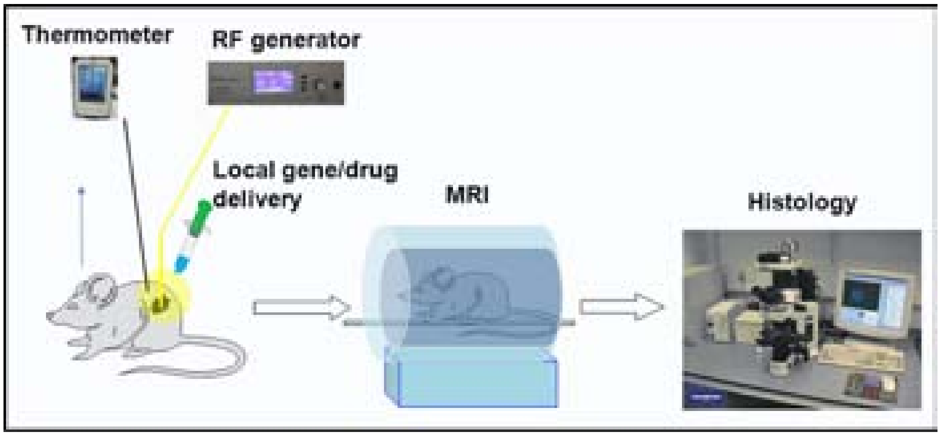

Supplementary Figure S2: *In vivo* experiment set-up for RFH-enhanced gene therapy. The nude mouse bearing a human breast cancer was treated with RFH at 45°C and subjected to direct intratumoral gene administration. The reduction in average tumor size was examined by follow-up MRI and confirmed by subsequent histology examination.
